# Eating Crow

**Published:** 1884-06

**Authors:** 


### EATING CROW.

*The Origin of the Phrase that is Just Now of Such Interest.*

AN old farmer who lived somewhere on the Hudson, below Albany, was in the habit of taking a few summer boarders to eke out the earnings of the farm. Like most farmers who take summer boarders and have at the same time a convenient market for their produce, this thrifty successor of the Knickerbockers was accustomed to send all the best products of his farm and garden and the choicest butter from his dairy to market, often returning from town with inferior articles which he had purchased at a greatly reduced price, safely hidden away in his wagon box to be smuggled into the kitchen and palmed off upon the confiding boarders as home-grown produce. Finally some of the boarders began to grumble. They had boarded in the country before and knew well what fresh vegetables and berries, new-laid eggs and "grass" butter were, and were conscious of the fact that they were not getting what they were entitled to. To all their complaints the farmer returned answer, they were entirely "too pertickeler" that it was foolish and simple to pamper one's appetite; that ordinary food was best in the long run, and winding up invariably with the remark; "I kin eat anything; I kin eat a crow." This last remark was repeated so often that it made an impression on one of the boarders, who, being out shooting one day, and having popped over a crow, determined to put the gastronomic abilities of his host to the test. He carried the bird home, had the cook dress it and gave her instructions to cook it for dinner. Then fearful that the hardy farmer might have a stomach even for such a dish and so make good his boast, he slipped into the kitchen while the bird was cooking, and seasoned it liberally with Scotch snuff. In time the dish was sent to the table and the boarder placed it before the host with the remark; "Now you have steadily proclaimed your ability to eat a crow. Here is one cooked to a turn. Try it." The farmer was somewhat taken back, but had too much pluck to acknowledge himself beaten without a trial. He accordingly attacked the dish with with the remark: "I kin do it." At the second bite he repeated: "I kin eat crow," and as he suddenly suspended the operation of cutting the third mouthful and began to retreat toward the door, he added, "but dang me if I hanker arter it!"

